# Geometry for low-inertia aerosol capture: Lessons from fog-basking beetles

**DOI:** 10.1093/pnasnexus/pgae077

**Published:** 2024-02-15

**Authors:** Aida Shahrokhian, Fan Kiat Chan, Jiansheng Feng, Mattia Gazzola, Hunter King

**Affiliations:** School of Polymer Science and Polymer Engineering, University of Akron, Akron, OH 44303, USA; Mechanical Sciences and Engineering, University of Illinois at Urbana-Champaign, Urbana, IL 61801, USA; School of Polymer Science and Polymer Engineering, University of Akron, Akron, OH 44303, USA; Mechanical Sciences and Engineering, University of Illinois at Urbana-Champaign, Urbana, IL 61801, USA; National Center for Supercomputing Applications, University of Illinois at Urbana-Champaign, Urbana, IL 61801, USA; Carl R. Woese Institute for Genomic Biology, University of Illinois at Urbana-Champaign, Urbana, IL 61801, USA; Physics, Rutgers University—Camden, Camden, NJ 08103, USA; Center for Computational and Comparative Biology, Rutgers University—Camden, Camden, NJ 08103, USA

**Keywords:** atmospheric water harvesting, bioinspired engineering, fluid dynamics

## Abstract

Water in the form of windborne fog droplets supports life in many coastal arid regions, where natural selection has driven nontrivial physical adaptation toward its separation and collection. For two species of Namib desert beetle whose body geometry makes for a poor filter, subtle modifications in shape and texture have been previously associated with improved performance by facilitating water drainage from its collecting surface. However, little is known about the relevance of these modifications to the flow physics that underlies droplets’ impaction in the first place. We find, through coupled experiments and simulations, that such alterations can produce large relative gains in water collection by encouraging droplets to “slip” toward targets at the millimetric scale, and by disrupting boundary and lubrication layer effects at the microscopic scale. Our results offer a lesson in biological fog collection and design principles for controlling particle separation beyond the specific case of fog-basking beetles.

**Significance Statement**

In the face of humanity’s increasing water insecurity, useful lessons in moisture management may be learned from organisms living in extreme climates. Two species of Namib desert beetle use their own bodies to extract water, in the form of fog, from the wind, and its dorsal features have inspired designs based on controlled wettability. Here, we revisit the role of these beetles’ surface topography from the fluid dynamics perspective of aerosol impaction. Experiments with natural samples, coupled to both artificial and computational analogs, reveal enhancement mechanisms enabled at disparate scales, which offer mechanistic insight to their possible role in biological fog capture and certain engineering applications.

## Introduction

With compounding effects of climate change and growing populations, humanity will need new technologies to satisfy its fundamental need for clean water. Where and when surface and ground sources are insufficient, the atmosphere itself can offer a supply not only as humidity, but as suspended liquid droplets in the form of clouds and fog. The potential for supporting human needs is hardly negligible: water in the atmosphere amounts to ∼3% of all freshwater, ∼13 quadrillion liters or ∼10,000 times our yearly global consumption (1).

In some regions, such as the Namib desert in Southwestern Africa, windborne fog is the primary source of life-sustaining water, and entire ecosystems have developed around mechanisms which separate it from the air (2, 3). When fog-laded air blows through vegetation, its slender elements present obstacles for the droplets to dodge as they are dragged along fluid streamlines. Their greater inertia causes droplets to lag behind sharp turns, and their occasional collisions lead to accumulation, enough to support both plants and animals which drink from wetted surfaces. Among the tenebrionid beetles that depend on fog harvesting in the Namib Desert, two species have adopted the unusual strategy of direct fog *basking*, using their own bodies instead of relying on vegetation (4). Climbing to the top of a dune on foggy mornings, clumsy in the cold (5), a beetle will lean its back into the wind, and as a solitary filtration element, wait for the microscopic droplets to accumulate and roll mouthward.

From the perspective of the beetle, there are two problems, and therefore two complementary directions for adaptation: how to get more droplets to collide and how to direct those accumulated to its mouth. Much has been written about possible adaptations in surface wettability to direct the transport of collected water and solve the second problem (6–27). Here, we focus on the first, significantly less explored issue, and hypothesize a dominant role of fundamental fluid mechanics in driving droplet impaction in the first place. We reinterpret local morphology and surface roughness as design features to manipulate flow streamlines and droplet trajectories, promoting collisions.

Fundamentals of the same flow physics guide the design of fog meshes (28, 29), which have proven a practical source of freshwater in rural communities, from the Andes to Macaronesia (30, 31). Similar to vegetation, they employ sparse (32) fibers to intercept droplets. Deposition efficiency $\eta_{D}$ (intercepted droplets over those initially headed toward collecting element) is controlled by the Stokes number *St* (droplet inertia over viscous drag), as empirically determined by (33)


(1) $$\eta_{D}∼\frac{St}{\left(\frac{\pi }{2}+St\right)}\;\text{with}\;St=\frac{2\rho_{w}}{9\mu_{a}}\left(\frac{ur^{2}}{R}\right),$$

where *R* is the target radius, *r* and $\rho_{w}$ are the droplet radius and density, $\mu_{a}$ is the dynamic viscosity of air, and *u* is the wind speed. This expression explains why slender elements are used: all else being equal, *St* increases linearly with decreasing target radius, and $\eta_{D}$ increases monotonically with *St*. It also shows why small droplets (St≪1) are hard to catch, and suggests that a beetle should struggle to catch any appreciable fog with its bulky body. Indeed, for a ∼1cm beetle in the challenging, but realistic conditions of fine fog (r∼2μm) and light breeze (u<2m/s), *St* becomes ≲0.01 and $\eta_{D}$ negligible.^a^ So, how could a beetle improve its chances of securing its primary moisture source?

The beetle cannot change the low *St* that limits its baseline efficiency without changing its size, which would affect a broad array of physiological functions. According to previous speculation, physical adaptation for fog basking may be embodied more modestly in the elytra surface properties alone (6). Subtle modification of their shape affect the local flow field, thus droplet trajectories and their tendency to be captured. In this investigation, we illustrate the role of morphology and surface roughness in fog collection by considering droplet dynamics up to first contact, thus *decoupling* our analysis from the subsequent effects of drainage. We find that, within the regime of extremely low efficiencies that characterize a fog-basking beetle, up to almost 400% water harvesting enhancement may be attained. Via experiments involving natural samples and computationally identified artificial analogs, we present mechanistic explanations for the relationship between shape, texture and impaction efficiency. Our results may serve as design principles for either boosting performance of aerosol capturing devices or inhibiting detrimental impaction on surface-modified aerodynamic elements.

## Results

### Experimental setup and collection efficiency

**Fig. 1.**
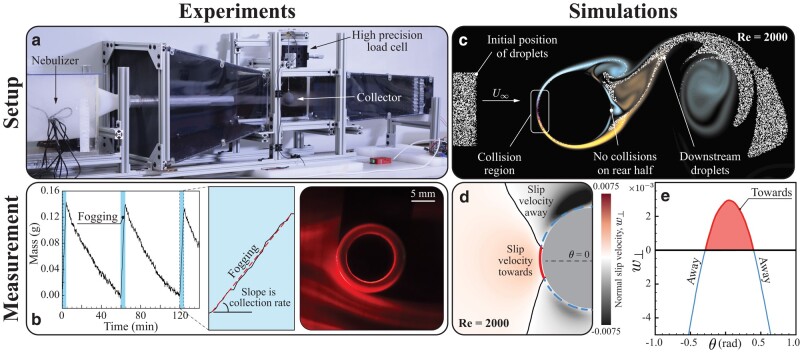
a) Table-top wind tunnel used for controlled fog collection experiments. A fog chamber equipped with nebulizers and an adjustable cover to regulate fog stream resistance. A direct current (DC) fan located downstream pulls air from the fog chamber and outside. The fog stream flows past the target, which hangs from a high-precision load cell to measure initial misting of the surface before coalescence. This is deliberate, to isolate impaction dynamics from those of drainage. b) Measured weight of a target (filtered to remove high-frequency noise) is plotted as a function of time. Shaded regions indicate fog generators in operation (4 min fogging periods). The slope of linear fit of a fogging period indicates the mass of water collected on the surface per second. Droplet pathlines around a cylindrical target, illuminated here by laser sheet from below, show a degree of spatial inhomogeneity, while their average density over time remains constant. c) Simulation of flow with fog particles incident on a cylinder with Re=2,000, r=1μm droplet radius, and free stream velocity $U_{\infty }=2$ m/s. d) Time-averaged normal slip velocity field $w_{⊥}$ (thin black line away from the surface represents isocontour of $w_{⊥}=0$). We note here that the flow shedding in the cylinder’s posterior, as seen in panel c, affects the upstream flow, with diminishing effect toward θ=0, thus resulting in the slight asymmetry in the slip velocity contours. e) $w_{⊥}$ evaluated near the cylinder’s surface, along the dotted line of panel d, is integrated (red region above the *x*-axis) to form the metric *W*, a proxy of efficiency $\eta_{D}$.

A fog impaction wind tunnel (35) (Fig. 1a) is used to measure water collection efficiency of given targets at prescribed *St* and Reynolds number $Re=\frac{2\rho_{a}uR}{\mu_{a}}$. To approximate the elusive, finer range of natural fog, droplets (r=1.6±0.9μm) are generated by upstream nebulizers similar to that of Ref. (36) and channeled toward 3D printed targets through a straight tube of minimal wall thickness. The target hangs from a high-precision load cell positioned in the center of the test section, and a variable speed DC fan drives the flow at u∼2m/s. The load cell measures accumulation of fog on the target for 4 min. This short window is deliberately chosen to separate impaction from drainage effects, as previously mentioned and further discussed in later sections. High-frequency noise is removed from individual measurements by windowed averaging of load cell data, and robust statistics of their linear slopes are gathered by repeating cycles of drying and fogging (Fig. 1b). Experimental deposition efficiency $\eta_{D}$ is then defined with respect to measured quantities as


(2) $$\eta_{D}=\frac{\dot{m}}{\phi \times u\times A},$$

where $\dot{m}$ is the collection rate (g/s), *ϕ* is the fog density (${g/m}^{3}$), *u* is the flow speed (m/s), and *A* is the target frontal area ($m^{2}$).

Experimental *St* is calculated from the expression on the right in Eq. 1, using the average radius from the measured drop size distribution (see Methods). *St* is then precisely varied by tuning the target diameter alone, leaving flow and fog properties unchanged. Consequently, as there is negligible uncertainty in distinguishing data points along *St*, its error bars are omitted in all figures.

### Flow simulations and droplet slip

Complementary direct numerical simulations (37, 38) of the flow-collector system are coupled with the general Maxey–Riley model (39) to capture inertial droplet dynamics (see Methods). This approach accurately obtains droplet trajectories (40) up to approximately one discretization grid point (Δx=R/512) from the target interface, beyond which numerical convergence degrades (a generic problem not specific to our approach). Figure 1c shows a distribution of fog droplets before and after advection past a 2D circular cylinder at beetle-relevant scales (Re=2,000, St=0.003). Only a small portion of droplets, exclusively on the windward side, comes close to contact. Which of these droplets actually make contact, we expect to sensitively depend on their slip velocity^b^ ($\vec{w}=\vec{v}-\vec{u}$) in the proximity of the surface, where $\vec{v}$ is the droplet velocity and $\vec{u}$ that of the surrounding fluid. Thus, the slip velocity, a key and broadly employed marker in fluid dynamics, quantifies how much a particle will deflect from the flow streamline it is currently sitting on. Without slip, no contact with any surface is possible. Luckily for the beetle, this quantity is exactly zero only in the case of passive tracers, i.e. massless, idealized particles that perfectly follow the flow. Figure 1d shows $w_{⊥}$, the field of average slip velocity normal to the interface, and its value along the dotted blue line is plotted in Fig. 1e as function of the angle *θ* from the horizontal axis. Reflecting experimental evidence and intuition, $w_{⊥}$ is greater near the stagnation point, where the sharply diverging streamlines are most likely to cause particles to drift. This suggests the practical use of $w_{⊥}$ in identifying collector regions where the probability of droplet impact is high, and, as we will see, to estimate the deposition efficiency $\eta_{D}$.

### Shape perturbation and efficiency estimate from simulations

**Fig. 2.**
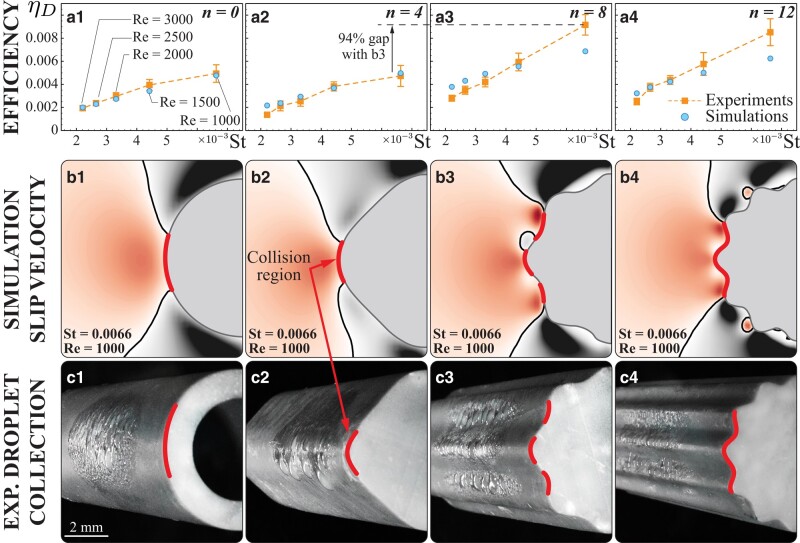
a) Integrating $W=\int w_{⊥}\;d\theta$ over the range where $w_{⊥}>0$ is positive, we fit the efficiency metric $\eta_{D}=aW^{b}$. The parameters *a* and *b* are determined using data *exclusively* from the smooth (n=0) cylinder, and then fixed throughout (a=0.069,b=0.41). As can be seen, the obtained relation allows us to map values of *W* from simulations into efficiencies $\eta_{D}$, even beyond the smooth cylinder case. Indeed, numerical predictions are shown to capture experimental trends across collectors and *St*, which was spanned in experiment by varying the nominal target diameter *L* from 7.5 to 22 mm. b) Color-coded normal component of average slip velocity fields of droplets, computed from simulated flows past smooth and wavy 2D cylindrical targets, evaluated for Re=1,000, St=0.0066. Black contours represent $w_{⊥}=0$, and the red outline represents the region in which radial slip velocity $w_{⊥}$ is positive. For all the cylinders, we observe slight asymmetry in the contours resulting from downstream flow shedding (e.g. region of negative $w_{⊥}$ near stagnation point in b3). Indeed, at these Reynolds numbers the flow is unsteady, leading to time varying $w_{⊥}$. While we time average the $w_{⊥}$ fields, slight asymmetries naturally persist. Longer time averages may mitigate these effects, although at significant additional computational costs. c) Water accumulated on the surface after 4 min of fogging period indicates range of collection, for the same *Re*, *St* (L=7.5mm). The collision region is in qualitative agreement with simulation results (regions where $w_{⊥}>0$).

Noting that $w_{⊥}$ depends on the flow curvature, we can already suspect that target morphology modulations could be used to locally inject sharp turns and induce droplet collisions. In Fig. 2, we then consider four cylindrical targets with sinusoidal perturbations added to their cross-sectional outlines, with wavenumbers $n=\frac{2\pi R}{\lambda }=$ 0, 4, 8, and 12. Across a range of conditions, we experimentally find that local curvature modulation indeed affects $\eta_{D}$, as seen, moving left to right along Fig. 2a, in the upward shift and steepening of the orange curves. The relationship with increasing wavenumber is nonmonotonic, with a steep gain of ∼94% between 4<n<8, and saturation afterwards. While the shape perturbations introduce additional surface area, the trend in efficiency clearly does not follow proportionally with its monotonic increase with *n* (see Methods), as one may expect if the collection mechanism was condensation rather than impaction (as previously observed in Ref. (35)). We note that absolute efficiencies over the explored range of *St* are small (up to 1%), an order of magnitude smaller than those optimized in mesh design. While this may seem negligible in some engineering contexts, it is hardly so for the beetle! Indeed, its overall scale forces the beetle to operate at highly unfavorable St<0.01 (unlike man-made meshes) and a fraction of percent gain in deposition efficiency may mean the difference between life and death for an organism with no other means of obtaining water.

We turn back to simulations for physical insight into the specific mechanism that enables these observed gains. Based on the intuition that large $w_{⊥}$ and surface extent over which $w_{⊥}>0$ both contribute to increasing collisions, we propose the proxy metric $W=\int w_{⊥}\;d\theta$, for $w_{⊥}>0$, which can be easily evaluated from simulated flows. Under the assumption that *W* captures the macroscopic flow mechanism underlying actual deposition efficiency $\eta_{D}$, we build the mapping $\eta_{D}=\phi (W)=aW^{b}$, an arbitrary functional form chosen for simplicity with two free parameters. Here, *a* and *b* are calibrated *once*, for the smooth cylinder case. Calibration proceeds as follows. Flow simulations spanning the *St* range of Fig. 2a1 are performed, and based on these, associated *W* are evaluated. Then, the parameters *a* and *b* are determined so as to map values of *W* to the corresponding, experimentally measured efficiencies $\eta_{D}$, after which *a* and *b* are fixed and used as is throughout. This procedure relates flow-droplet dynamics (up to contact) with collection efficiency, while it allows us to bypass the explicit consideration of final contact processes (i.e. long-range forces associated with surface chemistry and lubrication). Figure 2a1 illustrates the close comparison between simulations and experiments for n=0. Figure 2a2–a4 illustrates instead how the mapping $\eta_{D}=aW^{b}$ obtained for the smooth cylinder (a=0.069,b=0.41) generalizes across *Re*, *St*, and wavenumbers (n=4,8,12), predicting experimental deposition efficiencies. A mechanistic justification of the mapping is also seen in the qualitative agreement between predicted collision regions, where $w_{⊥}>0$ (red profiles in Fig. 2b), and observed collection regions (Figs. 2c and S1 for droplet pathlines).

### A step toward rational design

**Fig. 3.**
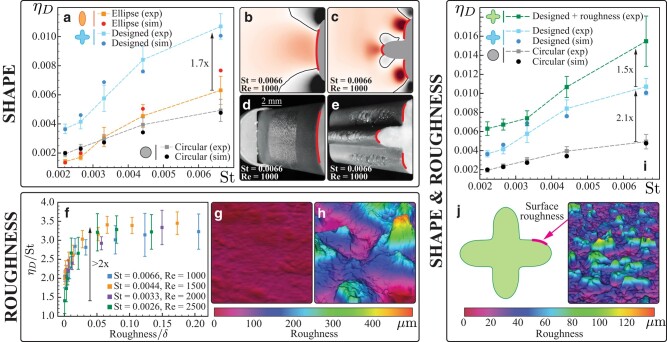
Shape. a) Deposition efficiency of elliptical, cylindrical, and designed collectors. b and c) Normal slip velocity field, $w_{⊥}$, around elliptical and designed collectors. Water collected on targets of elliptical d) and designed e) cross-section after 4 min of fogging. Collection in both cases occur at the positive slip velocity regions. Roughness. f) Deposition efficiency of roughened cylinders. *St* and *Re* numbers were altered by varying cylinder diameter. Data collapses when deposition efficiency is scaled by *St* and roughness by laminar boundary layer thickness, *δ*. g) Minimum and h) maximum roughness on the cylindrical targets, obtained by optical profilometry. Average equivalent particle diameter was used as the measure of roughness. Shape and roughness. i) Cumulative effects of macroscopic morphology (designed) and microscopic texture enhances the efficiency to up to 3.6× (designed + roughness) compared to the equivalent smooth cylinder. j) Designed target is covered with ∼100  μm rough coating (roughness profile shown on right).

With numerical tools in hand, we set out to computationally guide the rational design of target shapes, so as to improve fog collection while testing our slip modulation hypothesis. We start by introducing an approximately flat profile perpendicular to the flow, in the form of an elliptical cylinder (Fig. 3b and d). This has the effect of both retaining sharp streamline curvatures (as in the circular cylinder) and increasing the area of positive $w_{⊥}$. Naively, one would expect this shape modification to favor droplet capture. Nonetheless, the quantitative inspection of $w_{⊥}$ reveals that while the region of $w_{⊥}>0$ is indeed enlarged (Fig. 3b), the slip magnitude $\left|w_{⊥}\right|$ over this region is reduced, rendering $\eta_{D}$ comparable to the smooth circular cylinder, as confirmed experimentally across a range of Re/St in Fig. 3a. To increase $w_{⊥}$ in magnitude as well as area, we introduce a protruding rounded-nose at the stagnation point of the elliptical collector, as shown in Fig. 3c and e. Past its own stagnation region, the nose streamlines the incoming flow, thus reaccelerating the droplets along its surface toward the flat regions of the collector, with the consequence of producing two more stagnation points. While derived ad hoc, the result is instructive: this morphological alteration increases the range and magnitude of favorable slip velocity, enhancing impact probability and thus $\eta_{D}$, which is indeed seen to improve by up to ∼1.7× (blue in Fig. 3a) compared to the naively chosen elliptical cylinder.

Our metric thus reliably correlates with collection efficiency by accounting for droplet inertia in the target proximity, as determined by the upstream flow field. This allows us to accurately identify regions of high-probability impact. It also underscores a design principle based on “inertial traps,” whereby streamlined surfaces are interrupted by high curvature features, causing fluid to alternate between undisturbed accelerations and sudden turns. This manifests in multiple stagnation points where particles’ and fluid elements’ trajectories are separated, creating the necessary conditions for collision.

### Independent role of roughness

Experiments with smooth cylinders reveal that a second level of design, no longer at the macroscale, might be additionally relevant. From high-speed imaging near the stagnation point, we see that many droplets which make apparent collision with the target continue to roll or slide along the surface before eventually reentraining with the flow (Fig. S2). This behavior may be attributed to a complex competition between droplet surface tension, lubrication, and boundary layer effects. We then hypothesize that surface manipulation at a compatible, microscopic scale may interfere with these dynamics, countering sliding, and improving collection. We test this hypothesis by varying roughness in experiments. We note that roughness also affects wettability of the target surface, but that wettability plays no direct role in impaction and thus has no effect on measured efficiencies, as previously reported in Ref. (35), and as we will see in the next section. We employ sandpaper of different grit sizes to obtain circular cylinders of surface roughness varying from 1 to 250μm (by average equivalent particle diameter—Figs. 3g, h, and S3), and test them in the wind tunnel. Data are collapsed in Fig. 3f, where efficiency is scaled with the Stokes number *St*, and roughness with the laminar boundary layer thickness *δ*, estimated through the Blasius equation $\delta =\frac{5D}{\sqrt{Re}}$, where *D* is the cylinder diameter.^c^ As can be seen, upon increasing surface roughness, and for all cases, deposition efficiency first rapidly increases to then plateau, more than doubling collection.

Finally, because of separation of scales, the effects of macroscopic and microscopic design may be approximately additive, presenting the opportunity to combine them and further increase collection. Indeed, as can be appreciated in Fig. 3j, combined effects of macroscopic shape design (2.1×) and subsequent roughening (1.5×) leads to a cumulative efficiency increase of up to ∼3.6×.

### Beetle specimen analysis

**Fig. 4.**
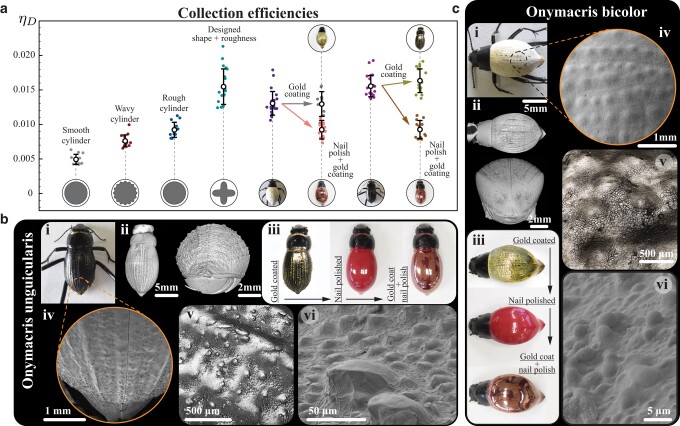
a) Deposition efficiency of the fog-basking beetles and 2D collectors of similar diameter (St=0.0066). b) *Onymacris unguicularis* and c) *Onymacris bicolor*, i) in natural state. ii) Micro-CT, iv) low-magnitude SEM, and v) optical profilometry images of the beetles show the larger scale features on the beetles: a) parallel ridges of ∼200μm wide and ∼100μm high; b) shallow bumps of ∼300μm diameter and ∼80μm high. vi) Closeup SEM images, taken slightly off the midline in the center of the elytra, show microscopic texture: a) two distinct scales of bumps, ∼50 and ∼10μm; b) bumps of ∼1μm diameter. b and c-iii) Surface chemistry of the beetles was altered by disposition of a thin layer of gold (∼20nm). Multiple thin coats of nail polish were applied to remove the effects of microscopic roughness. The smooth beetles were again coated with gold to maintain similar surface chemistry.

Returning now to the subject of our inspiration, how might we determine that these design principles have been incorporated in the physical adaptations of actual fog-basking beetles? First, we can measure their performance against our synthetic analogs in the same experimental setup, and for the same flow conditions. The left side of Fig. 4a summarizes the effects of surface modulation on the performance of targets of equal nominal diameter (and St=0.0066), and the right side shows the performance of the two known fog-basking species, *O. bicolor* and *O. unguicularis*. Unadulterated specimens (Fig. 4a) show efficiencies ∼3 times that of a smooth cylinder of equal *St*, and comparable to the rationally designed (shape + roughness) target of Fig. 3j.

From Computed Tomography (micro-CT) scans and scanning electron microscope (SEM) images we can visualize the beetles’ shape and microscopic texture (Figs. 4b, c, S5, and S6). Black *O. unguicularis* exhibits subtle ridges parallel to the flow when in basking pose (4) (∼200μm) and scattered bumps of two distinct scales, ∼50μm (Fig. 4b,v) and ∼10μm (Fig. 4b,vi). White-bodied *O. bicolor* has shallow bumps of ∼400μm (Fig. 4c,iv and v) and small protrusions of ∼1μm (Fig. 4c,vi). We note that while in our artificial samples we could engineer a sharp distinction between macroscopic *shape* (>100μm), which influences the flow field at distances greater than a droplet diameter from the target, and microscopic *texture* (<100μm), relevant to impact dynamics thereafter, the biological system presents a spectrum of scales. However, we can still test the consistency of our hypotheses by altering microscale effects (<100μm) in two ways. In one case, the beetle is sputter coated with a nanoscopic gold film which preserves the finest features while creating a uniform surface chemistry of high wettability. In the other, nail polish is carefully applied until observable features disappear, and then gold is coated to match the surface chemistry of the previous case (Fig. 4b and c,iii). In support of our thesis that pure fluid mechanics, rather than wettability patterns, dominates droplet impaction, the gold film alone causes no significant change in $\eta_{D}$ (Fig. 4a). Smoothing, however, causes $\eta_{D}$ to drop by factor of ∼2 for *O. unguicularis* and ∼1.5 for *O. bicolor* (Fig. 4a). This result is consistent with our experiments with artificial analogs, and illustrates the functional role of the beetle’s surface geometric features for fog harvesting.

### Where does surface transport come in?

Our study has focused on clarifying fluid dynamic principles gleaned from fog-basking beetles, and in doing so, has put aside the issue of drainage. However, its relevance demands an expanded discussion.

Despite its influence on biomimetic design, the notion of patterning wettability to control transport of collected water (6) does not appear to have been part of the evolutionary strategy of fog-basking beetles, which are in fact uniformly waxy (42). This does not mean that drainage and its related aspects of wettability and coalescence are not relevant. Indeed, in the continuous performance of fog capturing devices, they can play a critical role, as thoroughly illustrated previously (43, 44). However, as demonstrated in this study, they are not the sole players and cannot be related to collection efficiency in isolation. For instance, a poorly draining, fine fog mesh can clog its pores, diverting flow away from subsequent collection. Similarly, coalescence of droplets impacted on a slender target will predictably change its effective geometry, thus reducing *St* and $\eta_{D}$, as has been elegantly demonstrated by Refs. (34, 45). Enhancements in continuous performance seen in studies with slender fibers at higher *St* than our case, but with similarly varying roughness, surface chemistry, and cross-section shape (13, 14, 46) follow from the coupled effects of both flow physics and drainage dynamics. Our intent here has been to tease them apart for mechanistic insight.

## Conclusion

Our study then reframes, in purely fluid dynamic terms, the morphology of fog-basking beetles as possible adaptations for droplet separation. In this context, we uncover a two-scale strategy—to form “inertial traps” out of flow curvature at the macroscopic scale and to disturb droplet sliding at the microscopic scale. Toward application in improved fog harvesting, we demonstrate their additive effect on performance, and rational design principles. Further, we offer computational, predictive metrics to evaluate target shapes’ propensity for droplet collection.

Physical adaptation can be driven by conditions of greatest risk to survivability—for organisms dependent on direct fog basking, risk to its water access occurs when transient fog is fine and/or wind is slow. These conditions correlate with increased demand in general, when humidity is low (droplets shrink as they travel to drier locations). Increasing a poor baseline deposition efficiency by harnessing physical mechanisms tapped by natural selection could, in principle, lead to improved water collection when it is most desperately needed. Implications of the relationship between surface morphology and inertial impaction broadly extend across biological systems which depend on fog (3) or pollen (47, 48) capture, and engineering contexts, where impaction can be utilized for particle separation (49) or, alternatively, where surface modifications intended for aerodynamic benefit (50) can exacerbate unwanted capture such as icing (51).

## Methods

### Apparatus

#### Aerosol generation

Droplets of average diameter ∼2μm were generated using ultrasonic nebulizers (OT-SMG02, DC24V, 500 mA) submersed in DI water upstream in a chamber (Fig. 1a). The water level in the fog chamber was kept constant to sustain homogeneous droplet size distribution and production rate, using a closed-loop control system between a pressure sensor and solenoid valve which refills water to a pressure set point.

To measure droplet size distributions, cellulose acetate (CA) electrospun nanofibers (0.87±0.14μm in diameter) were used. The fibers were located at the outlet of the fog chamber with the same conditions as the experiments. The collision of microdroplets on the fibers were imaged by a high-speed camera (Phantom VEO 410, lens InfiniProbe TS-160). Directly after impaction, the diameters were measured in ImageJ, eliminating chance of evaporation or absorption of the droplets. Droplet size distributions are reported for this apparatus in Ref. (35) and are reproduced in comparison with those of Ref. (36) in the Supplementary material.

#### Aerosol delivery

The generated droplets were delivered to the target in the middle of the test section, through a funnel and a straight tube with minimal thickness (∼0.7mm) to reduce downstream disturbances. Uniform flow and fog column (without defects arising from introducing fog to the flow) was achieved by matching the flow speed between fog outlet and surrounding air. A DC fan at the end of the wind tunnel draw air for the both sources while the resistance of the pathways were tuned.

Area contraction ratio from the opening of the tunnel to test section was set at 4:1 (linear ratio 2:1). Flow in the tunnel was held constant for all experiments. Velocity across the width of the test section, measured with hot wire anemometer (HHF1001A, OMEGA Engineering Inc.) was 1.93±0.09m/s, and fluctuated approximately 3% over time at its center. Its profile can be found in the Supplementary material for Ref. (35).

Fog density *ϕ* was calculated from the flow speed *v*, the rate of water loss from the reservoir ${\dot{m}}_{\mathrm{res}}$, and the cross-sectional area of the tube *A*:


(3) $$\phi =\frac{-{\dot{m}}_{\mathrm{res}}}{vA}.$$

Water loss from the reservoir ${\dot{m}}_{\mathrm{res}}$ was measured via pressure sensor at its base and was found to maintain a constant value as long as the water level remained within the range set by the control loop. *ϕ* was assumed to be uniform within the fog column.

#### Accumulation measurements

The collectors were hung at the center of the test section from a sensitive load cell (FUTEK LSB200, precision 0.01 g) by a stainless-steel rod. To eliminate the axial force caused by drag, a Teflon rod with one point contact was assembled at the leeward side of the connecting rod. The temperature of the laboratory was recorded throughout the experiments, and to cancel the effects of temperature fluctuations on droplet size or loadcell output signal, only data recorded between 20.5 and 21.5 °C were presented.

To assure robust statistics, the experiments were executed in cycles of fogging and drying the surface (4 and 20 min, respectively) for at least 20 cycles. This frequency (24 min, $6.9\times 10^{-4}\;\mathrm{Hz}$) while being much longer than the vibrational noise generated by the flow and environment is much shorter than the frequency of the drift of the loadcell, aiding to achieve a desirable level of noise reduction from the raw signal. Here, the high-frequency flow fluctuation noise is eliminated by oversampling the load cell data (sampling frequency =40Hz) and applying moving average window (averaging every 5 s, 200 data points). The data representing the fogging period are then fitted with a linear function, whose slope indicates the deposition rate of the collector. Subsequently, the deposition efficiency of each cycle can be calculated by dividing deposition rate by the mass of water droplets that initially were headed toward the projected area of the target per second.

### Simulations

#### Flow field

Here, we briefly describe the governing equations and numerical technique used in our simulations. We consider a 2D solid body (i.e. target) immersed in an unbounded domain of incompressible viscous fluid. We denote the computational domain as $\Omega =\Omega_{\text{f}}\cup \Omega_{\text{s}}$, where $\Omega_{\text{f}}$ and $\Omega_{\text{s}}$ represent the fluid and solid domains, respectively, and define the interface between the fluid and the solid body as $\partial \Omega_{\text{s}}$. The flow is then described by the incompressible Navier–Stokes equation


(4) $$f\nabla \times u=0,\;\frac{\partial u}{\partial t}+\left(u\times \nabla \right)u=-\frac{1}{\rho }\nabla p+\nu \nabla^{2}u\;x\in \Omega \setminus \Omega_{\text{s}},$$

where *ρ*, *p*, u, and *ν* are the fluid density, pressure, velocity, and kinematic viscosity, respectively. We impose the no-slip boundary condition $u=u_{s}$ at $\partial \Omega_{s}$, where $u_{s}=0$ is the body velocity. The system of equations is solved in velocity–vorticity form using the remeshed vortex particle method combined with Brinkmann penalization (38). This method has been extensively validated across a range of fluid–structure interaction problems, from macroscale flow past bluff bodies and biological swimming (38, 52–55) to microscale viscous streaming responses from arbitrary shapes (56, 57). This numerical approach offers the advantages of simple computational representation of solid bodies with arbitrary shapes, a feature necessary for our study of morphological effects in fog harvesting, and inherent Eulerian–Lagragian representation, which allows for direct fog particle representation within our simulation framework.

#### Droplet trajectories

In order to include the fog droplets in our numerical simulation, we employ a one-way coupling method, where we capture only the fluid-to-particle effects and assume particle-to-fluid and particle-to-particle effects to be negligible, given their low volume fraction ($10^{-5}<\phi <10^{-4}$, as suggested in Ref. (58)). We then describe the particle’s equation of motion through general Maxey–Riley (MR) equation (39)


(5) $$\begin{array}{rl} m_{p}\frac{dV_{i}}{dt} & ={m_{f}\frac{Du_{i}}{Dt}|}_{Y(t)}\\ & \;-\frac{1}{2}m_{f}\frac{d}{dt}\left(V_{i}(t)-u_{i}[Y(t),t]-{\frac{1}{10}a^{2}\nabla^{2}u_{i}|}_{Y(t)}\right)\\ & \;-6\pi a\mu \left(V_{i}(t)-u_{i}\left[Y(t),t\right]-{\frac{1}{6}a^{2}\nabla^{2}u_{i}|}_{Y(t)}\right), \end{array}$$

where $m_{p}$ and $m_{f}$ are the particle and fluid mass, $V_{i}$ and $u_{i}$ are the particle velocity and fluid velocity at particle position Y(t), *a* is the particle radius, *μ* and *ν* are the dynamic and kinematic viscosity of the fluid, and *t* is the time.

Here, for simplicity, we assume a 2D problem where the effects of gravity and Basset history term (which does not make a significant contribution to integrated results (59), such as the dispersion of particles here) are excluded in our model. Therefore, on the right-hand side of the equation, we retain only the pressure gradient term, added mass term, and the viscous Stokes drag term, including the Faxen terms (terms with second-order derivatives), which we found to have insignificant effect in our simulation results. Nevertheless, we included them for completeness.

### Targets

#### Shape

Cylindrical targets were printed using a stereolithography 3D printer (Formlabs Form3, 25μm resolution). To conserve uniform surface energy and wetting properties of the collectors, only one type of resin (Formlabs GREY V4FLGPGR04) was used (static contact angle $\theta_{E}∼66^{\circ }$, measured on a flat surface).

Millimetric scale surface modifications were created by defining the cross-section using the following equation:


(6) r=R+k×Rcos(nθ),

where *n* determines the number of waves (0⩽n⩽16), *R* is the radius of equivalent smooth cylinder (1,000≤Re≤3,000), *k* is a constant establishing the amplitude of the waves (wavy collectors k=1/15, designed collectors k=2/5), and *r* is the radius of the surface at *θ* angle (0⩽θ⩽2π). Percentage increase in surface area for n=4,8, and 12 was 11, 43, and 91%, respectively, evaluated according to


(7) $$\int_{0}^{2\pi }\sqrt{1+{\left(kn\cos\;(n\theta )\right)}^{2}}\;d\theta -2\pi .$$

The equivalent ellipse geometries were generated by


(8) $${\left(\frac{x}{p}\right)}^{2}+{\left(\frac{y}{q}\right)}^{2}=R^{2},$$

where p=0.3×(1+k) and q=1+k (k=2/5).

#### Roughness

Commercially available silicon carbide (SiC) sandpaper sheets with grit ranging from 60 to 15,000 were used to generate rough surfaces (60 to 3,000 from 3 M, 5,000 to 15,000 from amazon). The SiC layer was carefully separated from the paper and then glued on the surface of the cylinders. The profile of the rough surfaces were then measured using IFM (Alicona Infinite Focus G5 Microscope, axial resolution 500nm). The roughness parameter was evaluated as averaged equivalent particle diameter.

#### Beetles

The dead beetles, *O. unguicularis* and *O. bicoloris*, were first dried and cleaned using acetone and DI water. To smooth the back morphology and evaluate the effect of surface features on collision efficiency, the beetles were coated with thin layers of nail polish until the features disappeared. The morphology of their elytra (before and after coating) was characterized by Micro-CT Scanner (Bruker Skyscan 1172, resolution 7μm), IFM (Alicona Infinite Focus G5 Microscope, axial resolution 500nm), and SEM (JEOL, sputter coated with gold for 20 s). The beetles (in natural state, coated with nail polish and sputter coated with gold) were positioned perpendicular to the direction of the flow in the wind tunnel, regardless of their natural basking position, to only evaluate their deposition efficiency with respect to their back morphology and variation in surface chemistry.

## Supplementary Material

pgae077_Supplementary_Data

## Data Availability

All data are contained within the manuscript.

## Notes

a While Eq. 1 is approximately valid only for St>1.1, more accurate predictions of efficiency for smaller *St* (34) also become negligible.

b Not to be confused with “slip” in a flow boundary condition at an interface with another medium.

c This relation, while referring to the flat plate, at leading order and near the windward edge, is valid for circular cylinders as well (41).

## Preprints

A preprint of this article is published at https://doi.org/10.21203/rs.3.rs-1397131/v1.
