# Correction to: Geometry for low-inertia aerosol capture: Lessons from fog-basking beetles

**DOI:** 10.1093/pnasnexus/pgae146

**Published:** 2024-04-09

This is a correction to: Aida Shahrokhian, Fan Kiat Chan, Jiansheng Feng, Mattia Gazzola, Hunter King, Geometry for low-inertia aerosol capture: Lessons from fog-basking beetles, PNAS Nexus, Volume 3, Issue 2, February 2024, pgae077, https://doi.org/10.1093/pnasnexus/pgae077

In the originally published version of this manuscript, in the originally published version of this manuscript the author Mattia Gazzola was inadvertently not listed as a corresponding author. This has been corrected in the paper.

