# Supplementary material for: Geometry for low-inertia aerosol capture: Lessons from fog-basking beetles
Source: PNAS Nexus. 2024 Feb 15;3(2):pgae077. doi: 10.1093/pnasnexus/pgae077 (PMC10903646; doi:10.1093/pnasnexus/pgae077)
Supplement: pgae077_Supplementary_Data [file pgae077_supplementary_data.pdf]

Supporting information for:

Geometry for low inertia aerosol capture: lessons from fog basking beetles

A. Shahrokhian, F. K. Chan, J. Feng, M. Gazzola & H. King

*Corresponding author email:

mgazzola@illinois.edu

h.king@rutgers.edu

This PDF file includes:

- Supplementary Figs. S1 to S6

Data availability. The data presented in this work is available upon request.

Code availability. Code for the analysis described in data analysis section and other analyses presented in this paper are available upon request.

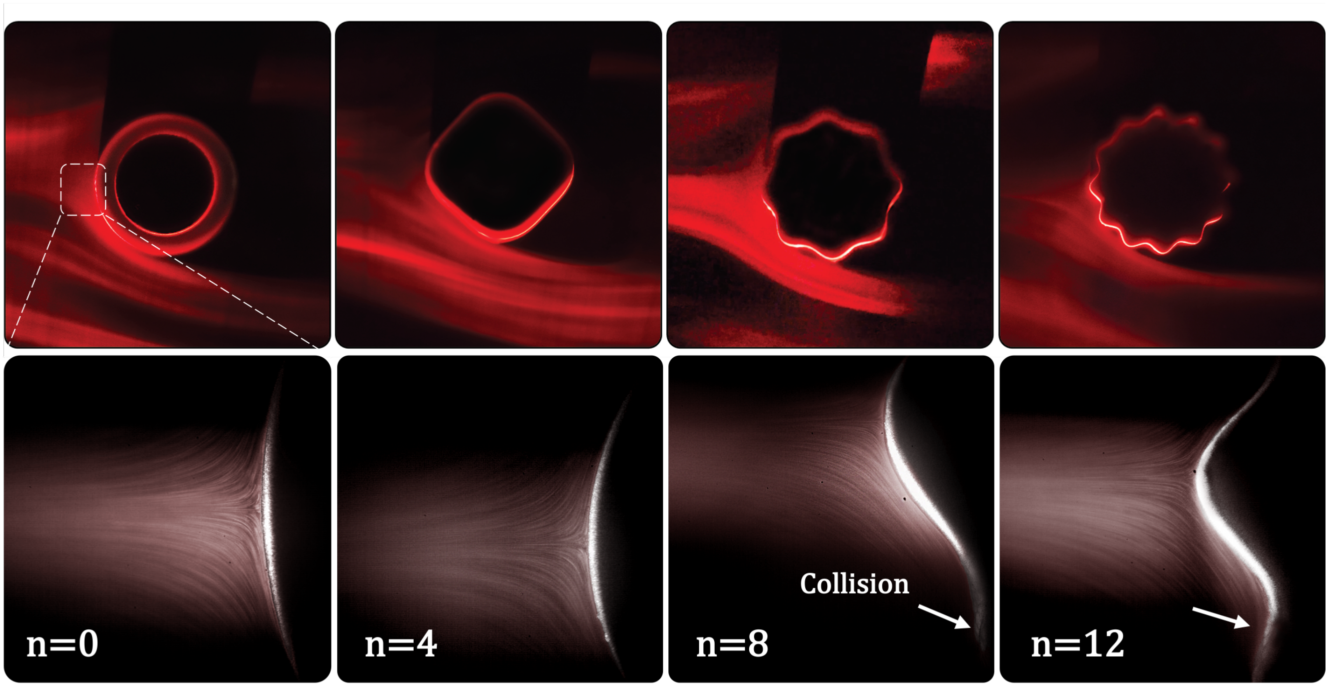

Fig.S 1: Top: Laser-illuminated flow around experimental targets; Bottom: Droplet pathlines near stagnation point, increasing the number of waves (n) from left to right. Collision of the droplets are observed in n=8 and 12 in early times.

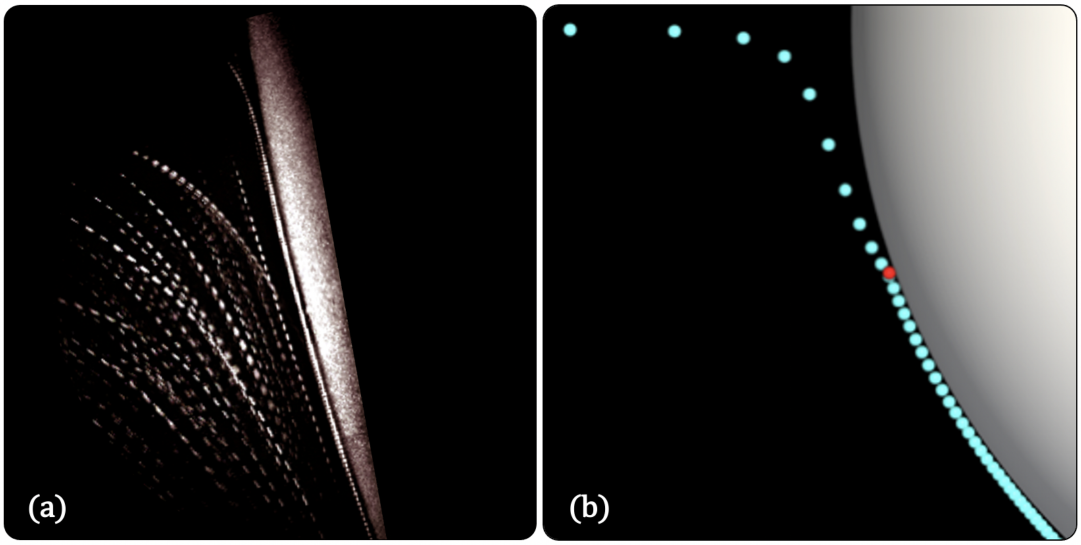

Fig.S 2: a) Droplet trajectories shows gliding behavior of droplets at very close distances to the surface, before reentering the free stream. Shown at intervals of $1ms$. b) Illustration of a single droplet approaching a surface from simulation. At distances within a particle diameter, simulations fail to distinguish the difference between a collision and near miss, due to finite resolution and unknowns in the hydrodynamic and adhesive forces between drop and surface. Trajectories including collision at the red dot and non-colliding path hugging the surface back to reentrainment span the uncertainty in determining impaction directly from simulations. For this reason, the empirical metric based on reliable values away from the surface was developed.

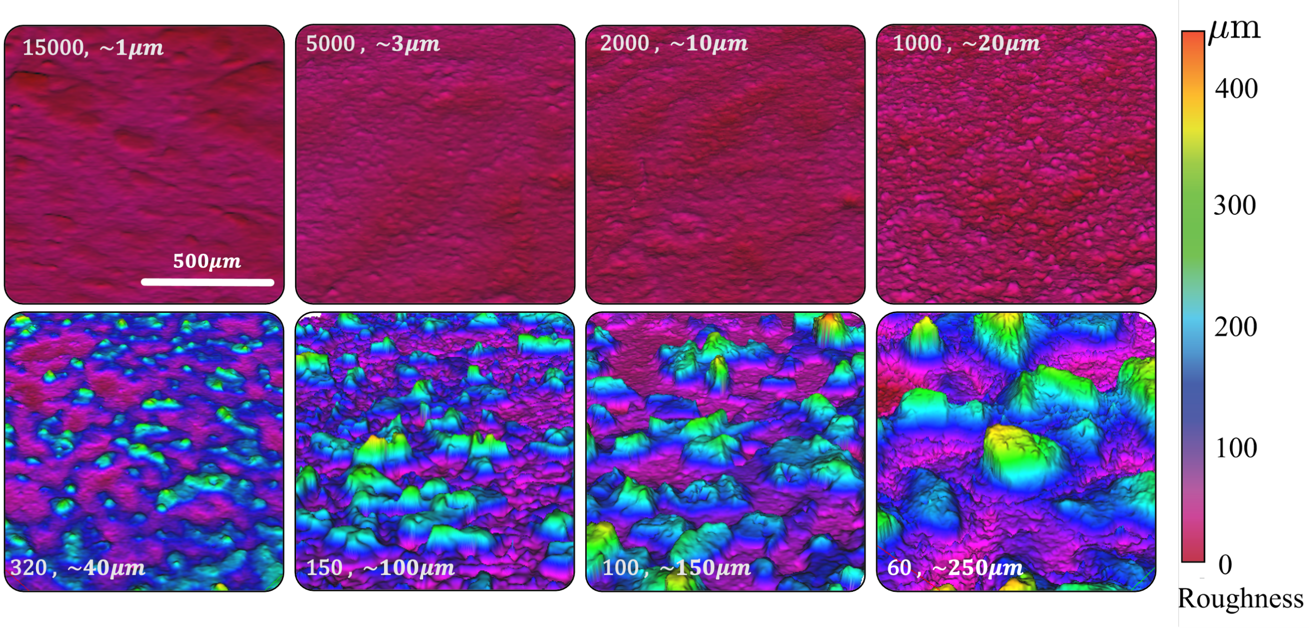

Fig.S 3: Top left: Minimum, bottom right: maximum roughness on the cylindrical targets, measured by optical profilometry. Averaged equivalent particle diameter for each grit (15000 to 60) are considered as the roughness of the surface (1 to 250 μm).

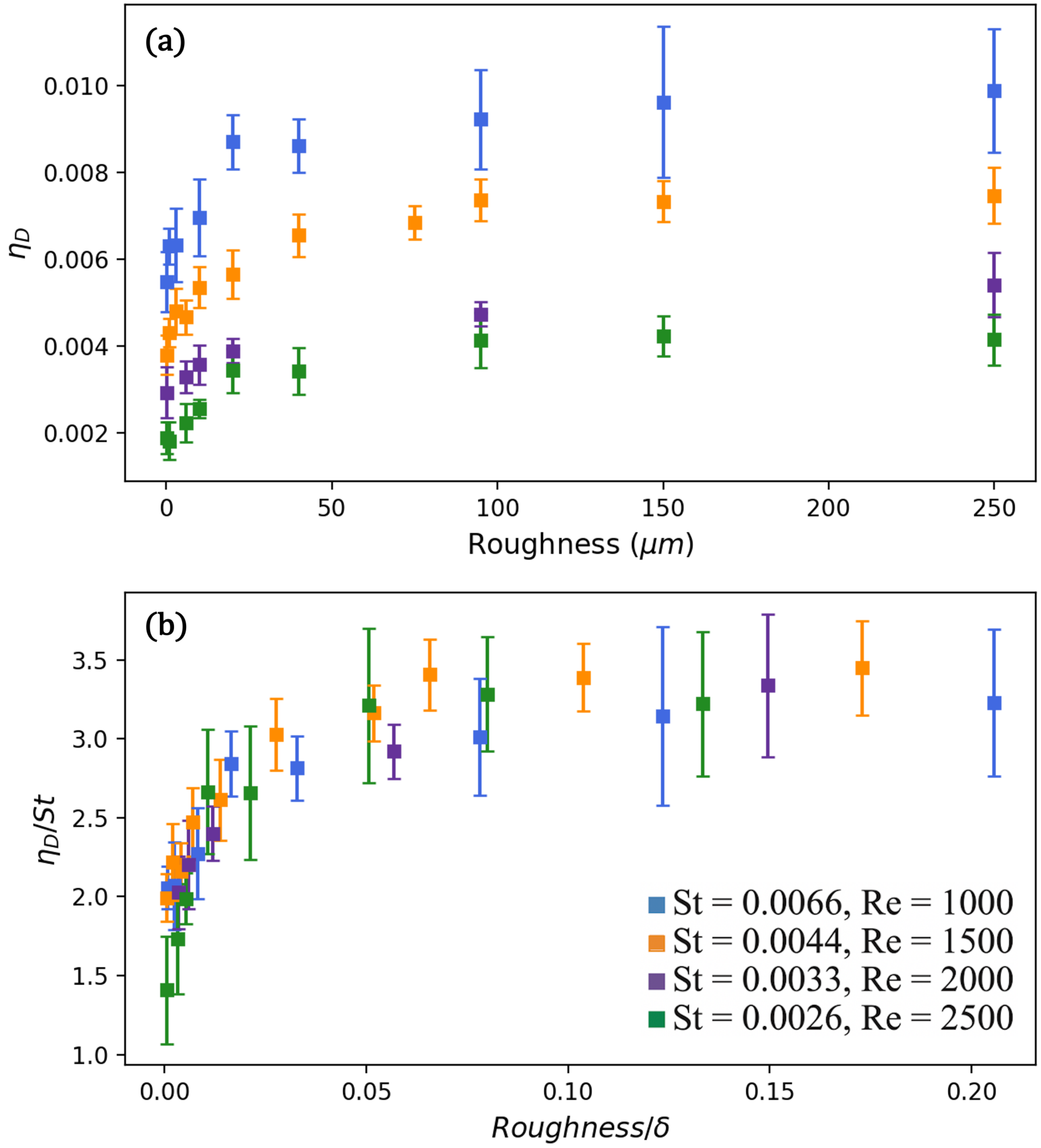

Fig.S 4: a) Deposition efficiency of roughened cylinders. St and Re numbers are altered by varying cylinder diameter. b) Data collapses, when deposition efficiency is scaled by St and roughness by laminar boundary layer thickness, δ.

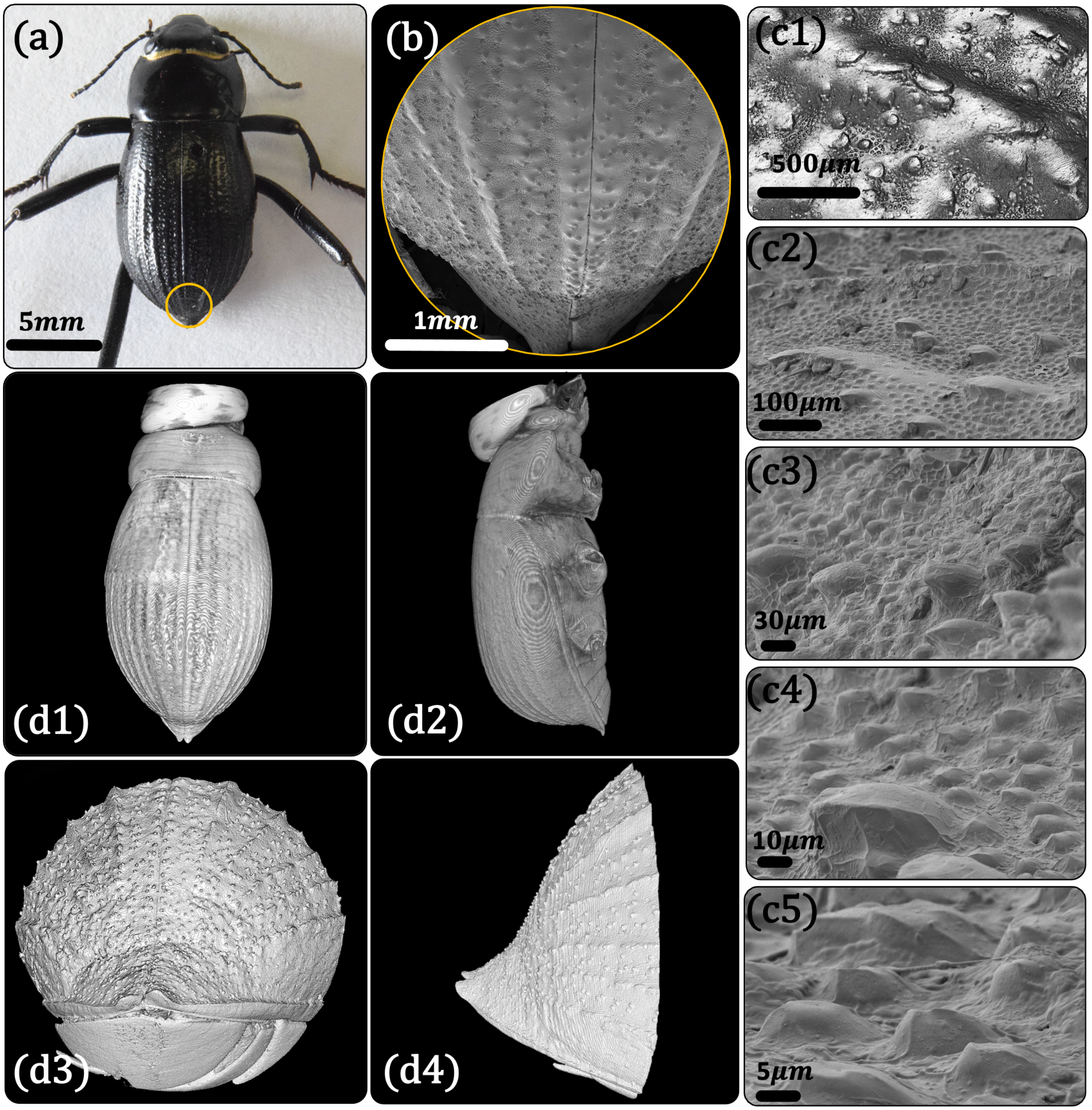

Fig.S 5: Shape and roughness of a) Onymacris unguicularis is characterized by b,c2-5) scanning electron microscopy, c1) optical profiler, and d1-4) micro CT scanner. Larger scale images (a,b,c1,d1-4) show parallel ridges of $\sim 200\mu m$ wide and $\sim 100\mu m$ high. Close up SEM pictures exhibit two distinct scales of bumps, $\sim 50\mu m$ and $\sim 10\mu m$.

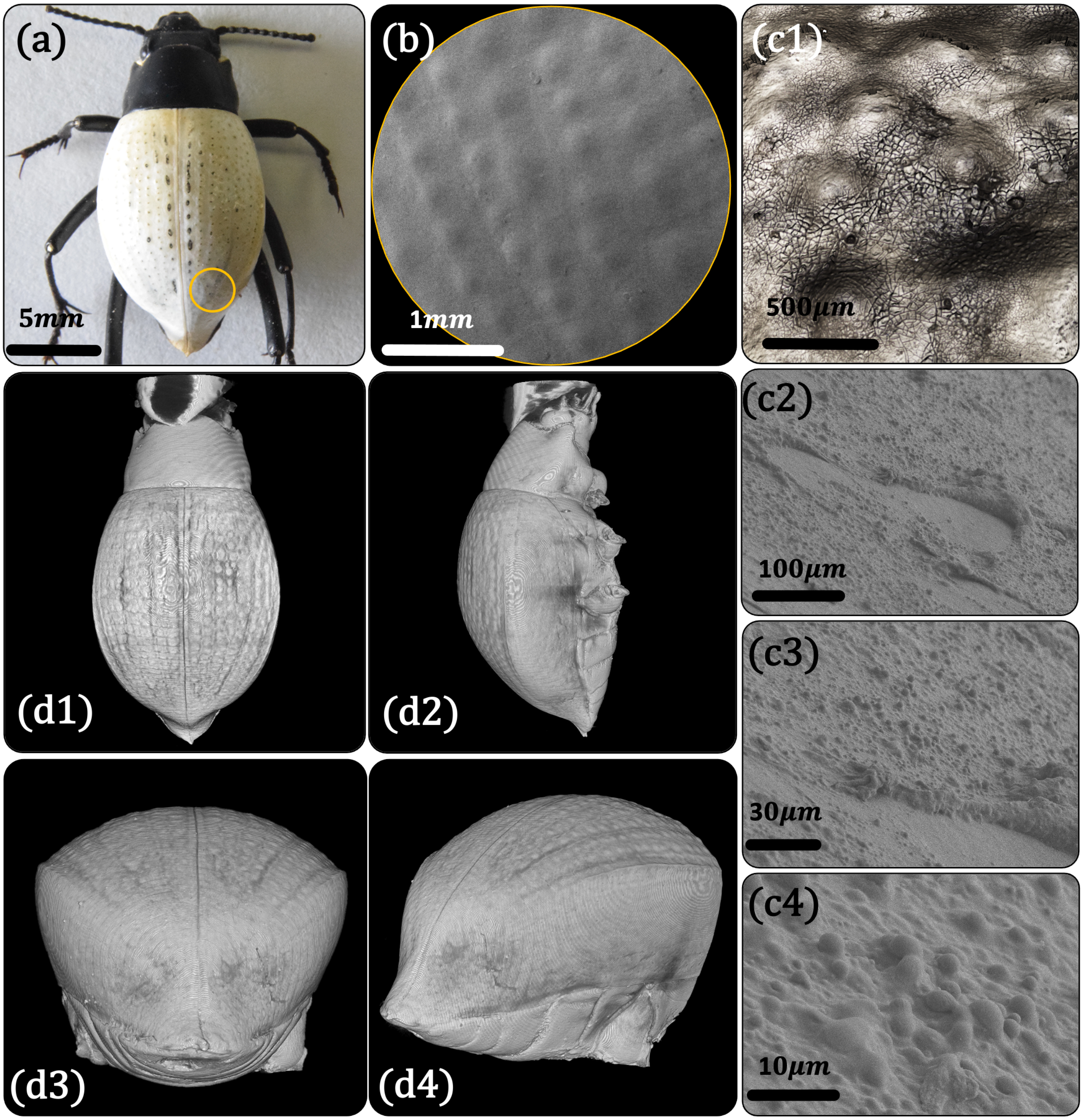

Fig.S 6: Shape and roughness of a) Onymacris bicolor is characterized by b,c2-4) scanning electron microscopy, c1) optical profiler, and d1-4) micro CT scanner. Larger scale images (a,b,c1,d1-4) show shallow bumps of $\sim 300\mu m$ diameter and $\sim 80\mu m$ high. Close up SEM pictures smaller bumps, approximately $1\mu m$.

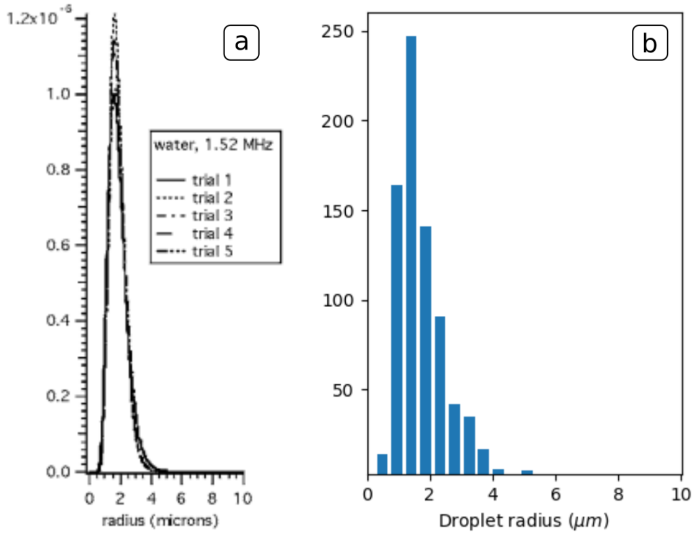

Fig.S 7: a) Droplet radius distribution for similar nebulizing fog source (from Ref. 36), obtained by Mie scattering, shows sharp peak at r=$2\mu m$. b) Radius distribution for our setup (from Ref. 35), measured by intercepting droplets on microscopically thin fibers and conventionally imaging, has similar peak and breadth.
